# A CT based radiomics nomogram for differentiation between focal-type autoimmune pancreatitis and pancreatic ductal adenocarcinoma

**DOI:** 10.3389/fonc.2023.979437

**Published:** 2023-03-01

**Authors:** Jia Lu, Nannan Jiang, Yuqing Zhang, Daowei Li

**Affiliations:** ^1^ Department of Radiology, The People’s Hospital of China Medical University and The People’s Hospital of Liaoning Province, Shenyang, China; ^2^ Department of Radiology, The People’s Hospital of Liaoning Province, Shenyang, China

**Keywords:** radiomics, focal-type autoimmune pancreatitis, pancreatic ductal adenocarcinoma, differential, machine learning

## Abstract

**Objectives:**

The purpose of this study was to develop and validate an CT-based radiomics nomogram for the preoperative differentiation of focal-type autoimmune pancreatitis from pancreatic ductal adenocarcinoma.

**Methods:**

96 patients with focal-type autoimmune pancreatitis and pancreatic ductal adenocarcinoma have been enrolled in the study (32 and 64 cases respectively). All cases have been confirmed by imaging, clinical follow-up and/or pathology. The imaging data were considered as: 70% training cohort and 30% test cohort. Pancreatic lesions have been manually delineated by two radiologists and image segmentation was performed to extract radiomic features from the CT images. Independent-sample T tests and LASSO regression were used for feature selection. The training cohort was classified using a variety of machine learning-based classifiers, and 5-fold cross-validation has been performed. The classification performance was evaluated using the test cohort. Multivariate logistic regression analysis was then used to develop a radiomics nomogram model, containing the CT findings and Rad-Score. Calibration curves have been plotted showing the agreement between the predicted and actual probabilities of the radiomics nomogram model. Different patients have been selected to test and evaluate the model prediction process. Finally, receiver operating characteristic curves and decision curves were plotted, and the radiomics nomogram model was compared with a single model to visually assess its diagnostic ability.

**Results:**

A total of 158 radiomics features were extracted from each image. 7 features were selected to construct the radiomics model, then a variety of classifiers were used for classification and multinomial logistic regression (MLR) was selected to be the optimal classifier. Combining CT findings with radiomics model, a prediction model based on CT findings and radiomics was finally obtained. The nomogram model showed a good sensitivity and specificity with AUCs of 0.87 and 0.83 in training and test cohorts, respectively. The areas under the curve and decision curve analysis showed that the radiomics nomogram model may provide better diagnostic performance than the single model and achieve greater clinical net benefits than the CT finding model and radiomics signature model individually.

**Conclusions:**

The CT image-based radiomics nomogram model can accurately distinguish between focal-type autoimmune pancreatitis and pancreatic ductal adenocarcinoma patients and provide additional clinical benefits.

## 1 Introduction

The concept of autoimmune pancreatitis (AIP) was first proposed by Yoshida et al. in 1995 (1). As a rare chronic disease, AIP usually presents as recurrent acute pancreatitis with abundant pathological lymphoplasmacytic infiltration (2, 3). The current study classifies AIP into two types: diffuse pancreatitis and focal pancreatitis (4). Focal-type autoimmune pancreatitis (fAIP) presents with segmental involvement of the pancreatic parenchyma, accounting for approximately 28-41% of AIP cases (5, 6). The imaging and clinical features of fAIP and pancreatic ductal adenocarcinoma (PDAC) are very similar, including focal or mass-like enlargement of the pancreas and obstructive jaundice, making their differential diagnosis very difficult. In addition, the treatment and prognosis of the two diseases vary widely. AIP is a benign fibro-inflammatory disease that responds to steroid therapy within one month in 90% of cases (7), whereas PDAC requires surgical resection to cure. Studies have shown that nearly 16% of cases of AIP are misdiagnosed as PDAC and undergo unnecessary pancreatectomy, with approximately 5-21% of cases undergoing pancreatectomy being ultimately confirmed as AIP. Currently, the only reference standard for the differential diagnosis of fAIP from PDAC is post-operative histology. The imaging examination is lacking clear reference standards for definitive diagnosis (7). Therefore, it is crucial to develop a non-invasive and effective methods to distinguish fAIP from PDAC preoperatively, enabling clinicians in the selection of appropriate treatment strategies.

As an emerging technology in the field of medical imaging, radiomics has provided a large amount of quantitative high-throughput information on radiographic images, helping to describe the tumor heterogeneity and the corresponding microenvironment (8). In this way, more predictive information can be obtained from medical imaging data than just the traditional visual interpretation (9), and provides a new way of approaching clinical diagnosis. In the field of abdominal radiology, radiomics techniques have been extensively studied, aiming to predict the tumor grade, survival and response to treatment, and to distinguish benign from malignant lesions. Therefore, it has the potential to be a non-invasive diagnostic method with performance close to biopsy. Some studies have applied this technique to pancreatic diseases (10–17), with a few studies reporting that the radiomic features extracted from enhanced CT images have certain value in the identification of AIP and PDAC. However, a more accurate integrative analysis of radiomics nomogram models to discriminate between fAIP and PDAC has not been fully developed.

Therefore, this study aims to develop and validate a non-invasive, reproducible and personalized radiomics-based nomogram method for preoperative identification of fAIP and PDAC based on contrast-enhanced CT images.

## 2 Materials and methods

### 2.1 Patients

**Figure 1:**
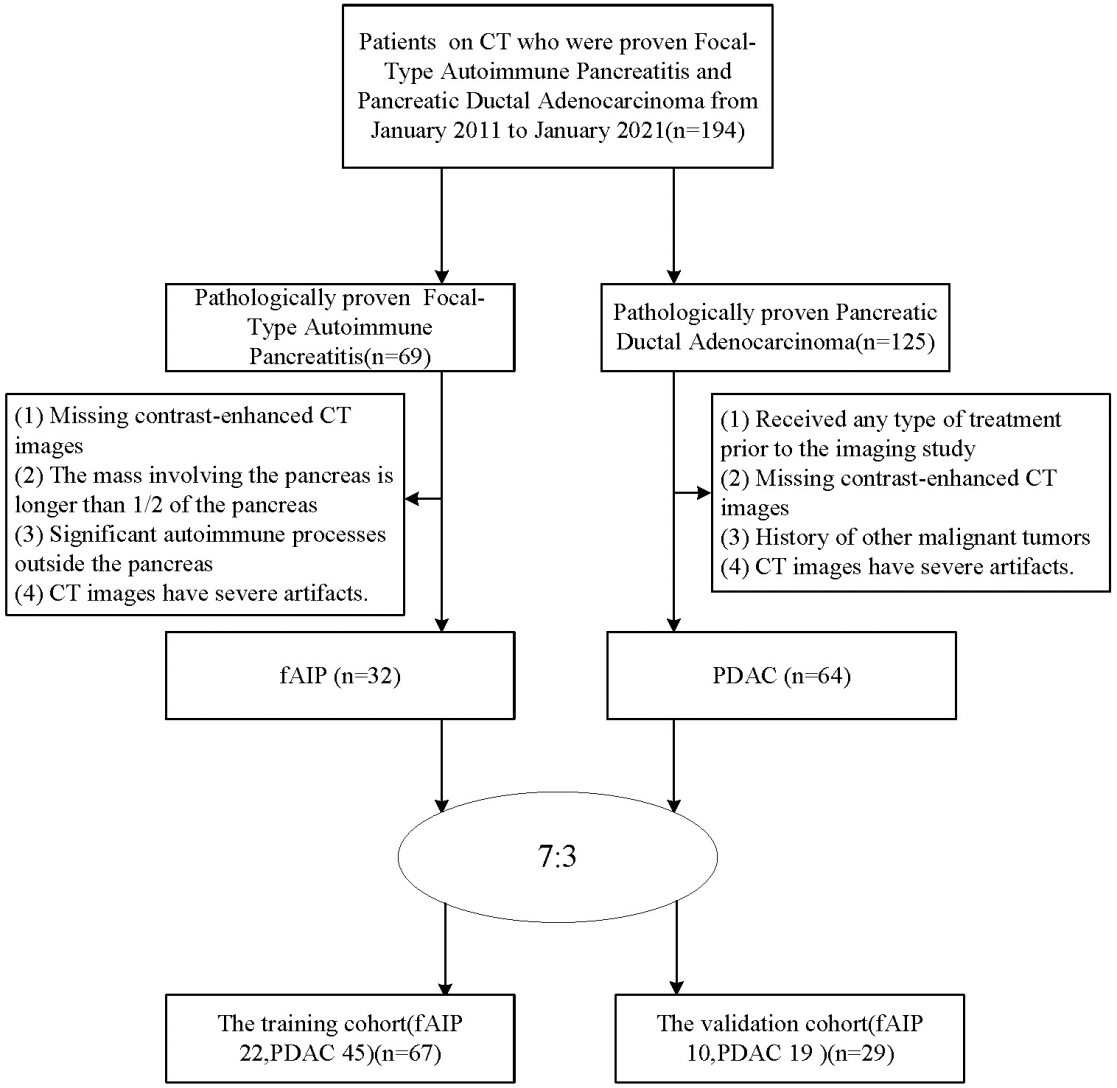
Patient Screening Flowchart.

The patients with fAIP between January 2011 and January 2021 in our hospital have been considered for this study. These patients were included according to the 2011 International Consensus Diagnostic Criteria (ICDC). The exclusion criteria were as follows (1): Contrast CT was not performed prior to steroid therapy or surgery; (2) The mass involving the pancreas is greater than 1/2 the length of the pancreas; (3) Significant autoimmune processes outside the pancreas, including sclerosing cholangitis, renal involvement, and retroperitoneal fibrosis, which may suggest fAIP; (4) CT images have severe artifacts. Finally, 32 patients with fAIP were included in our study (23 males, 9 females; mean ± SD: 60 ± 12.1 years; range: 43–82 years). Other patients from our hospital with PDAC pathologically confirmed between January 2017 and January 2022 were also considered. The exclusion criteria were as follows: (1) Received any type of treatment (radiation, chemotherapy, or chemoradiation) prior to the imaging study; (2) Enhanced CT scan was not performed within 1 month before surgery; (3) History of other malignancies; (4) CT images have severe artifacts. Finally, 64 patients with PDAC were included in our study (47 males, 17 females; mean ± SD: 60.1 ± 9.8 years; range: 40–88 years). Then, all patients were randomly divided into training cohort and test cohort at ratio of 7:3 (**Figure 1**). The clinical data were derived from medical records.

### 2.2 CT image acquisition

All patients were scanned with a 64-slice multidetector CT (SOMATOM, Definition AS+, Siemens, Forchheim, Germany). The parameters involved were as follows: 120 kVp; effective 180 mA; rotation time, 0.5 s; detector collimation, 32 × 1.2 mm; field of view, 350 × 350 mm; matrix, 512 × 512; section thickness, 5 mm; and reconstruction section thickness, 1.5 mm. All patients were required to fast for at least 6 hours and drink 500 to 800 mL water before the examination. Contrast-enhanced CT images were obtained after intravenous administration of nonionic contrast medium (Ultravist 300 mg I/mL; Bayer Schering Pharma AG, Berlin, Germany) at an injection rate of 2.5-3.0 mL/s using a power injector (1.5 mL/kg). The arterial phase images were scanned at 7 seconds after the attenuation value of abdominal aorta reached 100 Hounsfield units. The portal venous phase images were scanned at 40 seconds after the completion of the arterial phase scanning.

### 2.3 CT findings evaluation

The pancreas lesions CT images for each patient were independently evaluated and recorded in a blinded manner by two experienced abdominal radiologists (10 and 20 years of experience in the interpretation of abdominal radiology). If there was a discrepancy between the two radiologists for some cases, they would reach a consensus after reviewing the images again and consulting. CT quantitative parameters were based on the mean values recorded by two radiologists. Due to the different size of the lesions, the slices of patients are also different, each lesion is segmented into approximately 30 slices, each slice 1mm thick.

The CT images were analyzed, considering: (1) Location of lesions (head-neck and body-tail of the pancreas); (2) The size of the lesion (the largest diameter of the tumor in cross section) (3) Capsule-like rim; (4) Pancreatic atrophy (5) Biliary wall thickening(thickness≥3 mm); (6) Peripancreatic vascular involvement (invasion of the common hepatic artery, splenic artery and vein, gastroduodenal artery, superior mesenteric artery and vein, portal vein; the standard is vascular occlusion, stenosis, or more than half of the circumference is in contact with the tumor); (7) Regional lymph node swelling (Lymph node short diameter≥1 cm); (8) Abrupt bile duct cut-off; (9) Pancreatic ductal cut-off; (10) MPD dilatation upstream (Upstream PD expansion ≥ 5 mm).

### 2.4 Segmentation and feature extraction

**Figure 2:**
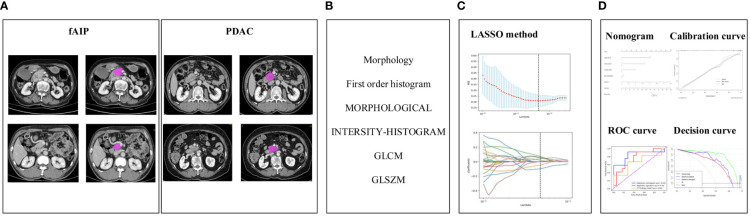
The research process including: **(A)** Data acquisition and segmentation; **(B)** Feature extraction; **(C)** Feature screening and **(D)** Radiomics nomogram construction and evaluation.

The construction process of the radiomics nomogram model is shown in **Figure 2**. The whole process includes: (A) Data acquisition and segmentation; (B) Feature extraction; (C) Feature screening and (D) Radiomics nomogram construction and evaluation.

#### 2.4.1 Image segmentation, feature extraction, and data preprocessing

We used the open-source software LIFEx (https://www.lifexsoft.org/index.php) to manually draw the three-dimensional volume of interest (VOI) of CT venous phase lesions. Particular care was taken to avoid the common bile duct and blood vessels while drawing the VOI. The segmentation process was performed by two experienced radiologists (10 and 20 years of experience in abdominal imaging), both of whom were blinded to the clinicopathological information of the patients, except the tumor location. The segmentation was finally completed with the consensus of the two radiologists.

LIFEx software is an open infrastructure software platform that flexibly supports common radiomics workflow tasks and is widely used in radiomics analysis. In our study, some parameters of LIFEx are as follows: In intensity discretization, nb of grep levels=400.0 and size of bins=10.0. In intensity Rescaling, min bound=-1000.0, max bound=3000.0 (18). We used LIFEx to extract 158 quantitative radiomics features. For each image, these features included six categories: morphological features, intensity features, grey-level cooccurrence matrix features (GLCM), grey-level distance zone-based features (GLDM), grey-level run-length matrix features (GLRLM), gray-level size zone matrix features (GLSZM), neighborhood grey tone difference-based features (NGTDM). The list of specific features we extracted is shown in **Table 1**. During data collection and image screening, we performed a normalization to ensure the reproducibility of our results.

**Table 1:** The extracted features using LIFEx toolbox.

Feature type	Feature name
MORPHOLOGICAL	MORPHOLOGICAL_Volume(IBSI : RNU0)
	MORPHOLOGICAL_ApproximateVolume(IBSI : YEKZ)
	MORPHOLOGICAL_voxelsCounting(IBSI : No)
	MORPHOLOGICAL_Compactness1(IBSI : SKGS)
	MORPHOLOGICAL_Compactness2(IBSI : BQWJ)
	MORPHOLOGICAL_SphericalDisproportion(IBSI : KRCK)
	MORPHOLOGICAL_Sphericity(IBSI : QCFX)
	MORPHOLOGICAL_Asphericity(IBSI:25C7)
	MORPHOLOGICAL_MaxValueCoordinates(IBSI : No)
	MORPHOLOGICAL_CenterOfMass(IBSI : No)
	MORPHOLOGICAL_WeightedCenterOfMass(IBSI : No)
	MORPHOLOGICAL_Hoc(IBSI : No)
	MORPHOLOGICAL_NormalizedHocRadiusRoi(IBSI : No)
	MORPHOLOGICAL_NormalizedHocRadiusSphere(IBSI : No)
	MORPHOLOGICAL_CentreOfMassShift(IBSI : KLMA)
	MORPHOLOGICAL_NormalizedHocRadiusRoi(IBSI : No)
	MORPHOLOGICAL_NormalizedHocRadiusSphere(IBSI : No)
	MORPHOLOGICAL_CentreOfMassShift(IBSI : KLMA)
	MORPHOLOGICAL_NormalizedHocRadiusRoi(IBSI : No)
	MORPHOLOGICAL_NormalizedHocRadiusSphere(IBSI : No)
INTENSITY	INTENSITY-BASED_Mean(HU)IBSI:Q4LE
	INTENSITY-BASED_Variance(HU)IBSI : ECT3
	INTENSITY-BASED_Skewness(HU)IBSI : KE2A
	INTENSITY-BASED_Kurtosis(HU)IBSI : IPH6
	INTENSITY-BASED_Median(HU)IBSI:Y12H
	INTENSITY-BASED_MinimumGreyLevel(HU)IBSI:1GSF
	INTENSITY-BASED_10thPercentile(HU)IBSI : QG58
	INTENSITY-BASED_25thPercentile(HU)IBSI : No
	INTENSITY-BASED_50thPercentile(HU)IBSI:Y12H
	INTENSITY-BASED_75thPercentile(HU)IBSI : No
	INTENSITY-BASED_90thPercentile(HU)IBSI:8DWT
	INTENSITY-BASED_StandardDeviation(HU)IBSI : No
	INTENSITY-BASED_MaximumGreyLevel(HU)IBSI:84IY
	INTENSITY-BASED_InterquartileRange(HU)IBSI : SALO
	INTENSITY-BASED_Range(HU)IBSI:2OJQ
	INTENSITY-BASED_MeanAbsoluteDeviation(HU)IBSI:4FUA
	INTENSITY-BASED_RobustMeanAbsoluteDeviation(HU)IBSI:1128
	INTENSITY-BASED_MedianAbsoluteDeviation(HU)IBSI:N72L
	INTENSITY-BASED_CoefficientOfVariation(HU)IBSI:7TET
	INTENSITY-BASED_QuartileCoefficientOfDispersion(HU)IBSI:9S40
	INTENSITY-BASED_AreaUnderCurveCsh(HU)IBSI : No
	INTENSITY-BASED_Energy(HU)IBSI:N8CA
	INTENSITY-BASED_RootMeanSquare(HU)IBSI:5ZWQ
	INTENSITY-BASED_TotalLesionGlycolysis(HU)IBSI : No
	INTENSITY-BASED_TotalCalciumScoreIBSI : No
	LOCAL_INTENSITY_BASED_IntensityPeakDiscretizedVolumeSought(0.5mL)(mL)IBSI : No
	LOCAL_INTENSITY_BASED_GlobalIntensityPeak(0.5mL)(HU)IBSI : No
	LOCAL_INTENSITY_BASED_IntensityPeakDiscretizedVolumeSought(1mL)(mL)IBSI : No
	LOCAL_INTENSITY_BASED_GlobalIntensityPeak(1mL)(HU)IBSI:0F91
	LOCAL_INTENSITY_BASED_LocalIntensityPeak(HU)IBSI : VJGA
	INTENSITY-BASED-RIM_Min(HU)IBSI : No
	INTENSITY-BASED-RIM_Mean(HU)IBSI : No
	INTENSITY-BASED-RIM_Stdev(HU)IBSI : No
	INTENSITY-BASED-RIM_Max(HU)IBSI : No
	INTENSITY-BASED-RIM_CountingVoxels(#vx)IBSI : No
	INTENSITY-BASED-RIM_ApproximateVolume(mL)IBSI : No
	INTENSITY-BASED-RIM_Sum(HU)IBSI : No
	INTENSITY-HISTOGRAM_IntensityHistogramMean(HU)IBSI:X6K6
	INTENSITY-HISTOGRAM_IntensityHistogramVariance(HU)IBSI : CH89
	INTENSITY-HISTOGRAM_IntensityHistogramSkewness(HU)IBSI:88K1
	INTENSITY-HISTOGRAM_IntensityHistogramKurtosis(HU)IBSI:C3I7
	INTENSITY-HISTOGRAM_IntensityHistogramMedian(HU)IBSI : WIFQ
	INTENSITY-HISTOGRAM_IntensityHistogramMinimumGreyLevel(HU)IBSI:1PR8
	INTENSITY-HISTOGRAM_IntensityHistogram10thPercentile(HU)IBSI : GPMT
	INTENSITY-HISTOGRAM_IntensityHistogram25thPercentile(HU)IBSI : No
	INTENSITY-HISTOGRAM_IntensityHistogram50thPercentile(HU)IBSI : No
	INTENSITY-HISTOGRAM_IntensityHistogram75thPercentile(HU)IBSI : No
	INTENSITY-HISTOGRAM_IntensityHistogram90thPercentile(HU)IBSI : OZ0C
	INTENSITY-HISTOGRAM_IntensityHistogramStandardDeviation(HU)IBSI : No
	INTENSITY-HISTOGRAM_IntensityHistogramMaximumGreyLevel(HU)IBSI:3NCY
	INTENSITY-HISTOGRAM_IntensityHistogramMode(HU)IBSI : AMMC
	INTENSITY-HISTOGRAM_IntensityHistogramInterquartileRange(HU)IBSI : WR0O
	INTENSITY-HISTOGRAM_IntensityHistogramRange(HU)IBSI:5Z3W
	INTENSITY-HISTOGRAM_IntensityHistogramMeanAbsoluteDeviation(HU)IBSI:D2ZX
	INTENSITY-HISTOGRAM_IntensityHistogramRobustMeanAbsoluteDeviation(HU)IBSI : WRZB
	INTENSITY-HISTOGRAM_IntensityHistogramMedianAbsoluteDeviation(HU)IBSI:4RNL
	INTENSITY-HISTOGRAM_IntensityHistogramCoefficientOfVariation(HU)IBSI : CWYJ
	INTENSITY-HISTOGRAM_IntensityHistogramQuartileCoefficientOfDispersion(HU)IBSI : SLWD
	INTENSITY-HISTOGRAM_IntensityHistogramEntropyLog10(HU)IBSI : No
	INTENSITY-HISTOGRAM_IntensityHistogramEntropyLog2(HU)IBSI : TLU2
	INTENSITY-HISTOGRAM_AreaUnderCurveCsh(HU)IBSI : No
	INTENSITY-HISTOGRAM_MaximumHistogramGradient(HU)IBSI:12CE
	INTENSITY-HISTOGRAM_MaximumHistogramGradientGreyLevel(HU)IBSI:8E6O
	INTENSITY-HISTOGRAM_MinimumHistogramGradient(HU)IBSI : VQB3
	INTENSITY-HISTOGRAM_MinimumHistogramGradientGreyLevel(HU)IBSI : RHQZ
	LOCAL_INTENSITY_HISTOGRAM_IntensityPeakDiscretizedVolumeSought(0.5mL)(mL)IBSI : No
	LOCAL_INTENSITY_HISTOGRAM_GlobalIntensityPeak(0.5mL)(HU)IBSI : No
	LOCAL_INTENSITY_HISTOGRAM_IntensityPeakDiscretizedVolumeSought(1mL)(mL)IBSI : No
	LOCAL_INTENSITY_HISTOGRAM_GlobalIntensityPeak(1mL)(HU)IBSI : No
	LOCAL_INTENSITY_HISTOGRAM_LocalIntensityPeak(HU)IBSI : No
	INTENSITY-HISTOGRAM-RIM_Min(HU)IBSI : No
	INTENSITY-HISTOGRAM-RIM_Mean(HU)IBSI : No
	INTENSITY-HISTOGRAM-RIM_Stdev(HU)IBSI : No
	INTENSITY-HISTOGRAM-RIM_Max(HU)IBSI : No
	INTENSITY-HISTOGRAM-RIM_CountingVoxels(#vx)IBSI : No
	INTENSITY-HISTOGRAM-RIM_ApproximateVolume(mL)IBSI : No
	INTENSITY-HISTOGRAM-RIM_Sum(HU)IBSI : No
GLCM	GLCM_JointMaximum(IBSI : GYBY)
	GLCM_JointAverage(IBSI:60VM)
	GLCM_JointVariance(IBSI : UR99)
	GLCM_JointEntropyLog2(IBSI : TU9B)
	GLCM_JointEntropyLog10(IBSI : No)
	GLCM_DifferenceAverage(IBSI : TF7R)
	GLCM_DifferenceVariance(IBSI:D3YU)
	GLCM_DifferenceEntropy(IBSI : NTRS)
	GLCM_SumAverage(IBSI : ZGXS)
	GLCM_SumVariance(IBSI : OEEB)
	GLCM_SumEntropy(IBSI:P6QZ)
	GLCM_AngularSecondMoment(IBSI:8ZQL)
	GLCM_Contrast(IBSI : ACUI)
	GLCM_Dissimilarity(IBSI:8S9J)
	GLCM_InverseDifference(IBSI : IB1Z)
	GLCM_NormalisedInverseDifference(IBSI : NDRX)
	GLCM_InverseDifferenceMoment(IBSI : WF0Z)
	GLCM_NormalisedInverseDifferenceMoment(IBSI:1QCO)
	GLCM_InverseVariance(IBSI:E8JP)
	GLCM_Correlation(IBSI : NI2N)
	GLCM_Autocorrelation(IBSI : QWB0)
	GLCM_ClusterTendency(IBSI : DG8W)
	GLCM_ClusterShade(IBSI:7NFM)
	GLCM_ClusterProminence(IBSI : AE86)
GLRLM	GLRLM_ShortRunsEmphasis(IBSI:22OV)
	GLRLM_LongRunsEmphasis(IBSI:W4KF)
	GLRLM_LowGreyLevelRunEmphasis(IBSI:V3SW)
	GLRLM_HighGreyLevelRunEmphasis(IBSI:G3QZ)
	GLRLM_ShortRunLowGreyLevelEmphasis(IBSI : HTZT)
	GLRLM_ShortRunHighGreyLevelEmphasis(IBSI : GD3A)
	GLRLM_LongRunLowGreyLevelEmphasis(IBSI : IVPO)
	GLRLM_LongRunHighGreyLevelEmphasis(IBSI:3KUM)
	GLRLM_GreyLevelNonUniformity(IBSI:R5YN)
	GLRLM_RunLengthNonUniformity(IBSI:W92Y)
	GLRLM_RunPercentage(IBSI:9ZK5)
NGTDM	NGTDM_Coarseness(IBSI : QCDE)
	NGTDM_Contrast(IBSI:65HE)
	NGTDM_Busyness(IBSI : NQ30)
	NGTDM_Complexity(IBSI : HDEZ)
	NGTDM_Strength(IBSI:1X9X)
GLSZM	GLSZM_SmallZoneEmphasis(IBSI:5QRC)
	GLSZM_LargeZoneEmphasis(IBSI:48P8)
	GLSZM_LowGrayLevelZoneEmphasis(IBSI : XMSY)
	GLSZM_HighGrayLevelZoneEmphasis(IBSI:5GN9)
	GLSZM_SmallZoneLowGreyLevelEmphasis(IBSI:5RAI)
	GLSZM_SmallZoneHighGreyLevelEmphasis(IBSI : HW1V)
	GLSZM_LargeZoneLowGreyLevelEmphasis(IBSI : YH51)
	GLSZM_LargeZoneHighGreyLevelEmphasis(IBSI:J17V)
	GLSZM_GreyLevelNonUniformity(IBSI : JNSA)
	GLSZM_NormalisedGreyLevelNonUniformity(IBSI:Y1RO)
	GLSZM_ZoneSizeNonUniformity(IBSI:4JP3)
	GLSZM_NormalisedZoneSizeNonUniformity(IBSI : VB3A)
	GLSZM_ZonePercentage(IBSI:P30P)
	GLSZM_GreyLevelVariance(IBSI : BYLV)
	GLSZM_ZoneSizeVariance(IBSI:3NSA)
	GLSZM_ZoneSizeEntropy(IBSI : GU8N)

#### 2.4.2 Intra- and inter-observer reliability

To assess inter-observer reliability, blinded two radiologists performed VOI segmentation. For intra-observer reliability, features were extracted twice by the first observer at a one-month interval. Reliability was calculated using the intraclass correlation coefficient (ICC). Radiomics signatures with both intra- and inter-observer ICC values greater than 0.75 (indicating excellent stability) were selected for follow-up investigations.

#### 2.4.3 Dimensionality reduction and feature selection

Feature selection consists of two steps: independent samples t-test and least absolute shrinkage and selection operator (LASSO) logistic regression algorithm. Regarding the selection of hyperparameters of the LASSO algorithm, after repeated training, we selected *alphas*=[0.001, 0.05, 50], and the final optimal *alpha* value was 0.04832; we selected *cv*=5, which was determined according to the amount of data, in order to ensure that the number of each sample set divided is more than 15 samples, thereby ensuring the stability of the model; *max_iter*=100000 is selected, to ensure that there are enough iterations for the model to complete the training. The other parameters and their values have been added to the additional file. Finally, each patient’s radiomics score (Rad-Score) was calculated using a linear combination of selected features weighted by the respective coefficients.

#### 2.4.4 Machine learning classifier selection

We analyzed the classification performance of the following four most used classifiers: Multivariate Logistic Regression (MLR), Random Forest (RF), Support Vector Machine (SVM), and Decision Tree (DT). These four classifiers were used to train the feature data in the training cohort. The 5-fold cross-validation method was used to ensure the stability and reliability of the training results, the classification performance was evaluated using the test cohort, and the hyperparameters of the four classifiers can be found in the additional file. To ensure that the number of samples in each data set divided is more than 15, and to ensure the training effect of the classifier, k = 5 has been empirically determined through the trial-and-error method (k range: 5–15, step size of 5) (19). To obtain the same percentage of patient status in both training and test datasets, in each training process, although the sample size of training is determined by the total amount of data, the sample size of the two types of data is equal.

### 2.5 Radiomics nomogram construction and evaluation

We developed combinatorial models combining CT findings and radiomic features. A radiomics nomogram was then generated from the above features by MLR, providing clinicians with the appropriate tool to differentiate between fAIP and PDAC in each patient. We then plotted a calibration curve for the nomogram, graphically showing the agreement between the predicted and actual probabilities of the radiomics nomogram, and presented the prediction process and results with two randomly selected patients and assessment. To further measure the predictive performance of the combined model, we used the receiver operating characteristic (ROC) area under the curve (AUC) to quantify the radiomics nomogram with 95% confidence interval (95% CI) and compared it to the single model. In order to ensure the consistency of the classifiers and then correctly evaluate the predictive ability of each model, we used the MLR classifier on both the CT findings model and the radiomics model for classification, depending on previous studies (20). Finally, the decision curves for the three models were plotted to assess the overall net benefit performance of the radiomics nomogram model.

### 2.6 Statistical analysis

All the statistical analyses were performed using R software (version 3.6.0, https://www.r-project.org) and Python (version 3.7.0, https://www.python.org). Continuous variables were expressed as mean ± standard deviation and compared by independent t-test with normal distribution or Mann-Whitney U test with abnormal distribution. Differences in categorical variables were analyzed by chi-square test or Fisher’s exact test. Multivariate logistic regression analysis was used to select independent predictors in the subjective CT findings model. Values with two-sided P < 0.05 were considered statistically significant.

## 3 Results

### 3.1 Clinical characteristics and CT findings model

The clinical characteristics of the patients with fAIP and PDAC are listed in **Table 2**, and the CT findings of patients are shown in **Table 3**. All clinical characteristics showed no significant difference between the fAIPs group and the PADCs group (P=0.325~0.873). CT images, including capsule-like rim, pancreatic atrophy, biliary wall thickening and vascular invasion, differed significantly between the two groups (P<0.05), indicating these features have a certain role in the diagnosis of fAIP and PDAC. There is no significant difference between groups in other characteristics (P>0.05).

**Table 2:** Clinical characteristics of patients.

Characteristics	fAIPs	PDACs	*P* value
	(n=32)	(n=64)	
Age (year), mean ± SD	60 ± 12.1	60.1 ± 9.8	0.635
Gender			0.873
Male	23	47	
Female	9	17	
Location			0.645
Head and neck	21	45	
Body and tail	11	19	
Maximum section diameter, mean ± SD	44.8 ± 16.7	41.6 ± 13.8	0.325

fAIPs, focal-type autoimmune pancreatitis; PDACs, pancreatic ductal adenocarcinoma; SD, standard deviation.

**Table 3:** CT findings of the patients.

Characteristics	fAIPs	PDACs	*P* value
	(n=32)	(n=64)	
Capsule-like rim	20	3	<0.001*
Regional lymph node swelling	7	25	0.094
Abrupt bile duct cut-off	2	12	0.104
Pancreatic atrophy	7	35	0.002*
Pancreatic ductal cut-off	4	19	0.064
Biliary wall thickening	17	4	<0.001*
Vascular invasion	2	51	<0.001*
MPD dilatation upstream(>5mm)	13	33	0.317

fAIPs, focal-type autoimmune pancreatitis; PDACs, pancreatic ductal adenocarcinoma; *P<0.05.

### 3.2 Radiomic signature construction and evaluation

**Figure 3:**
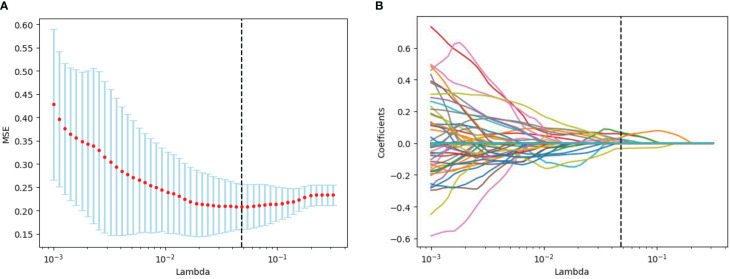
Radiomics feature screening by LASSO regression algorithm. **(A)** Plot of polynomial deviation versus λ. The red dots represented the mean deviation value for each model with a given λ, the vertical line was plotted at the best value by using the minimum criterion, where 7 features had non-zero coefficients. **(B)** Distribution of LASSO coefficients for radiological features. Each colored line represents the coefficient of each feature.

**Figure 4:**
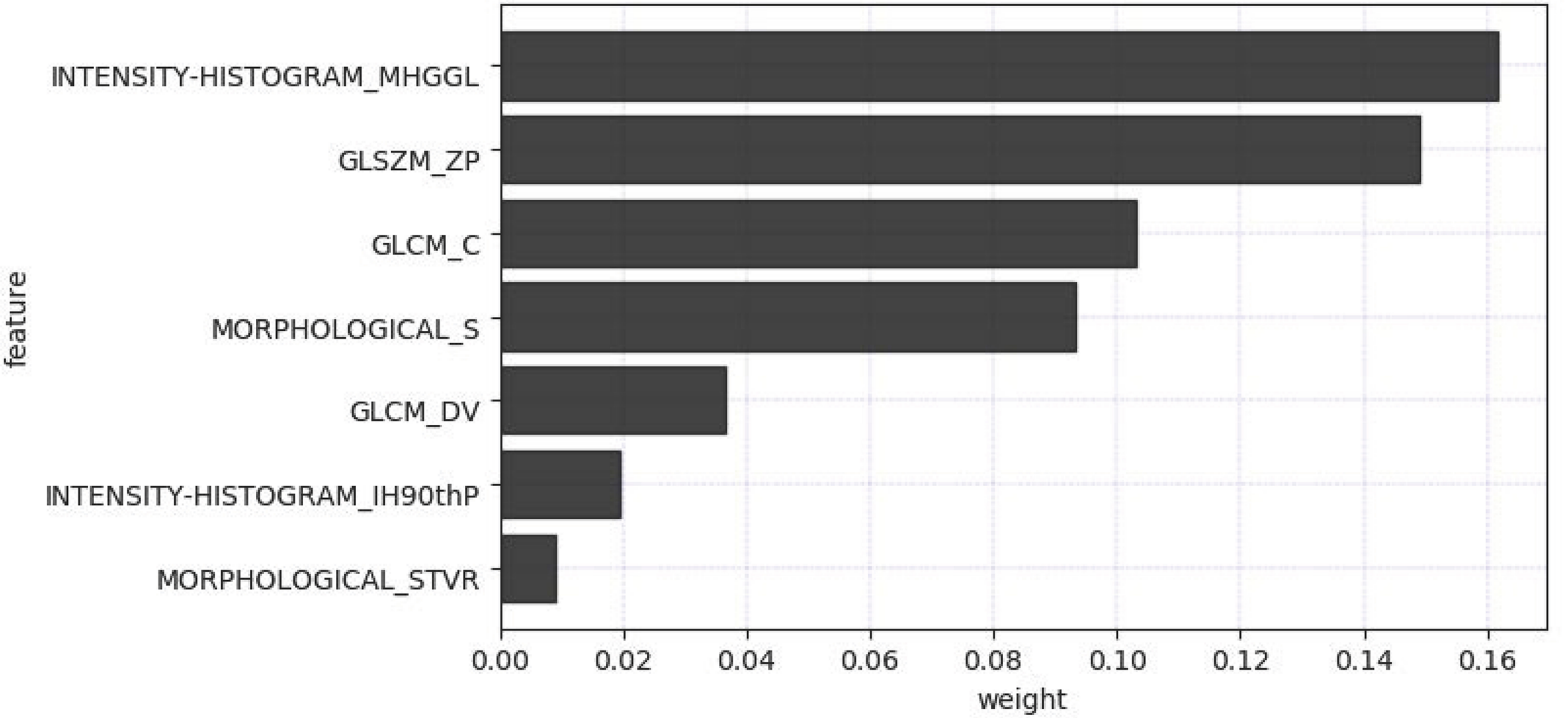
Filtered feature names and its weight performance.

The radiomics feature selection process was performed separately at various stages. Based on venous phase CT images, 38 features were initially extracted by independent samples t-test. After removing redundant features, 7 potential features were selected by the LASSO algorithm. Then, a multiparametric radiomics signature based on venous phase images was established (**Figure 3**), the final filter gets the feature name and its weight performance (**Figure 4**).

According to the feature data screened by the LASSO algorithm, different classifiers based on machine learning to classify the feature data were used, and 5-fold cross-validation was performed to ensure the stability of the classification results. The test cohort was then used to verify the performance of different classifiers, and obtain the classification results as shown in **Table 4**.

**Table 4:** Classification performance of different classifiers.

Classifier	Training cohort (n=67)				Test cohort (n=29)			
	ACC	AUC	Sensitivity	Specificity	ACC	AUC	Sensitivity	Specificity
MLR	0.72	0.73	0.95	0.97	0.71	0.76	0.89	0.93
RF	0.92	0.95	1.0	1.0	0.56	0.60	1.0	0.17
SVM	0.89	0.93	1.0	0.92	0.61	0.69	0.93	0.48
DT	0.69	0.71	0.95	0.90	0.65	0.70	0.87	0.72

MLR, Multivariate Logistic Regression; RF, Random Forest; SVM, Support Vector Machine; DT, Decision Tree; ACC, Accuracy; AUC, Area Under the Curve.

Specifically, the favorable radiomics signature can be expressed by Rad-score:


$$\begin{array}{l} Rad-Score=16.1747-\\ \left(0.009034*MORPHOLOGICAL\_SurfaceToVolumeRatio\right)+\left(0.093138*MORPHOLOGICAL\_Spehericity\right)\\ -\left(0.019595*INTENSITY-HISTOGRAM\_IntensityHistogram90thPercentile\right)-\\ \left(0.161556*INTENSITY-HISTOGRAM\_MaximumHistogramGradientGreyLevel\right)-\\ \left(0.036626*GLCM\_DifferenceVariance\right)+\left(0.103065*GLCM\_Correlation\right)-\\ \left(0.148918*GLSZM\_ZonePercentage\right) \end{array}$$

After the Rad-score calculation for the fAIPs group (median: -0.81; range: -2.50~-0.09) was significantly lower than that of the PDACs group (median: -0.34; range: -0.63~0.62). We tested both sets of data using an independent samples t-test and found p-values < 0.001 for both sets of data.

### 3.3 Radiomics nomogram construction and validation

**Figure 5:**
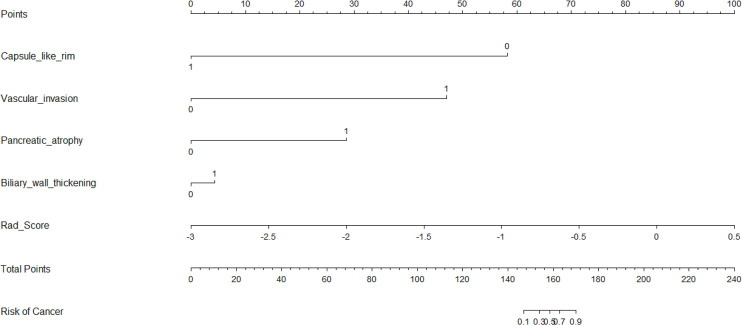
Nomogram for differentiating focal-type autoimmune pancreatitis (fAIPs) and pancreatic ductal adenocarcinoma (PDACs). The Capsule-like rim, Pancreatic atrophy, Biliary wall thickening, Vascular invasion and Rad-score were used for building the radiomics nomogram. Plotted the first scale “points” to identify points for each predictor. When the total points were calculated by adding the scores of these five predictors, the corresponding prediction probability was obtained at the last scale.

**Figure 6:**
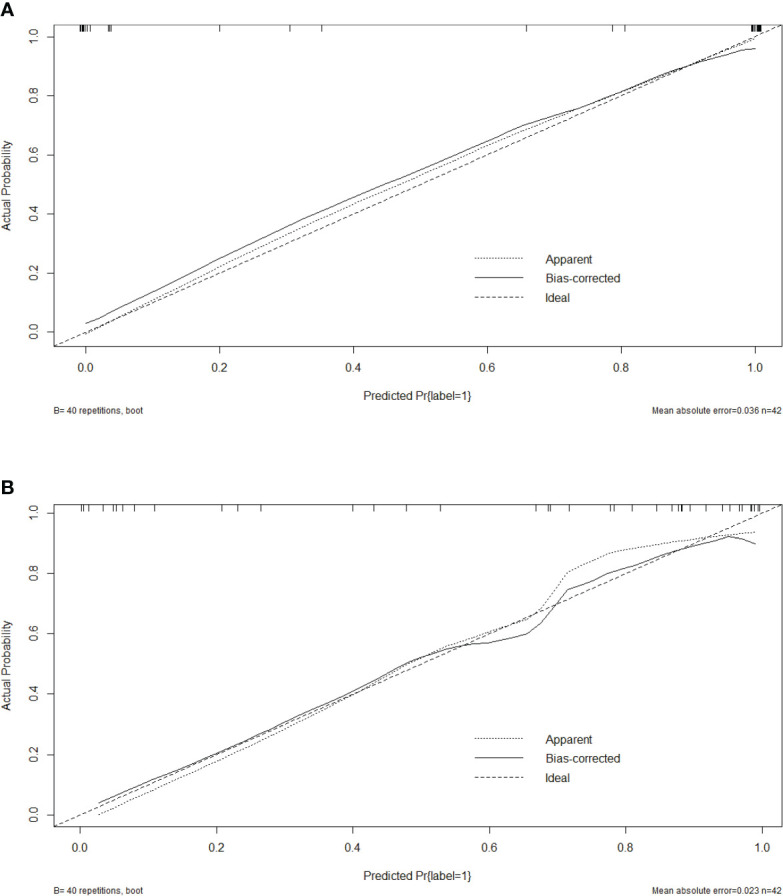
Calibration curves of the radiomics nomogram in training cohort **(A)** and test cohort **(B)**.

**Figure 7:**
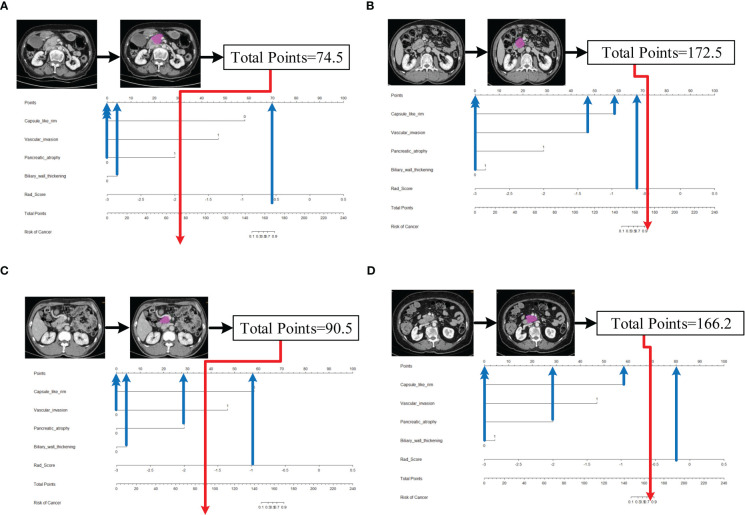
Schematic diagram of prediction flow of radiomics nomogram model. **(A)** after VOI delineating, image preprocessing, the value of total points was 74.5, which was calculated by the CT findings and Rad-Score. The result corresponded to <10% probability of a firm consistency. Thus, the patient’s disease was predicted to be fAIP, which was confirmed by ICDC. **(B)**The total points was 172.5, which corresponding to >90% probability of a firm consistency. Thus, the patient’s disease was predicted to be PDAC, which was confirmed in surgery. **(C)** The external validation data, the total points was 90.5. Thus, the patient’s disease was predicted to be fAIP, which was confirmed by ICDC. **(D)** The external validation data, the total points was 166.2. Thus, the patient’s disease was predicted to be PDAC, which was confirmed in surgery.

Five characteristics including the capsule-like rim, pancreatic atrophy, biliary wall thickening, vascular invasion and Rad-Score were included in the multivariate logistic regression analysis, and a combined model of radiomics nomogram was constructed (**Figure 5**). **Figure 6** shows that the nomogram calibration curve with good agreement between predictions and observations in both cohorts. In addition, we randomly selected two patients and used the radiomics nomogram model for prediction. The prediction process and results are shown in **Figures 7A, B**.

### 3.4 Comparison between different models

**Figure 8:**
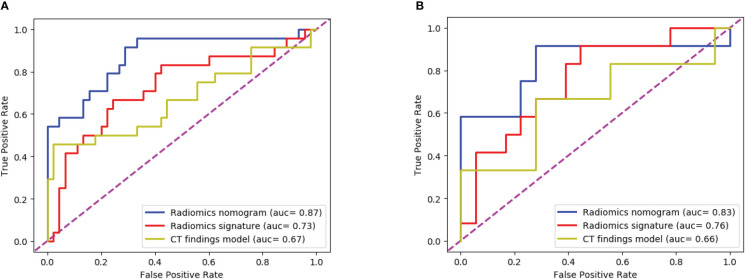
The ROC curves of the three models: **(A)** The training cohort; **(B)** The test cohort. The AUC values of the radiomics nomogram model were higher than that of the CT appearance model and the radiomics model in both training cohort and test cohort.

The ROC curves (**Figure 8**) analyzed the diagnostic ability of three different models in the training and test cohort. Radiomics nomogram showed the best diagnostic performance in both training (AUC = 0.87) and test cohort (AUC = 0.83), followed by radiomics signature (training cohort, AUC = 0.73; test cohort, AUC = 0.76). Both models outperformed the model based on CT findings in both the training (AUC = 0.67) (P < 0.05) and test cohorts (AUC = 0.66) (P < 0.05).

**Figure 9:**
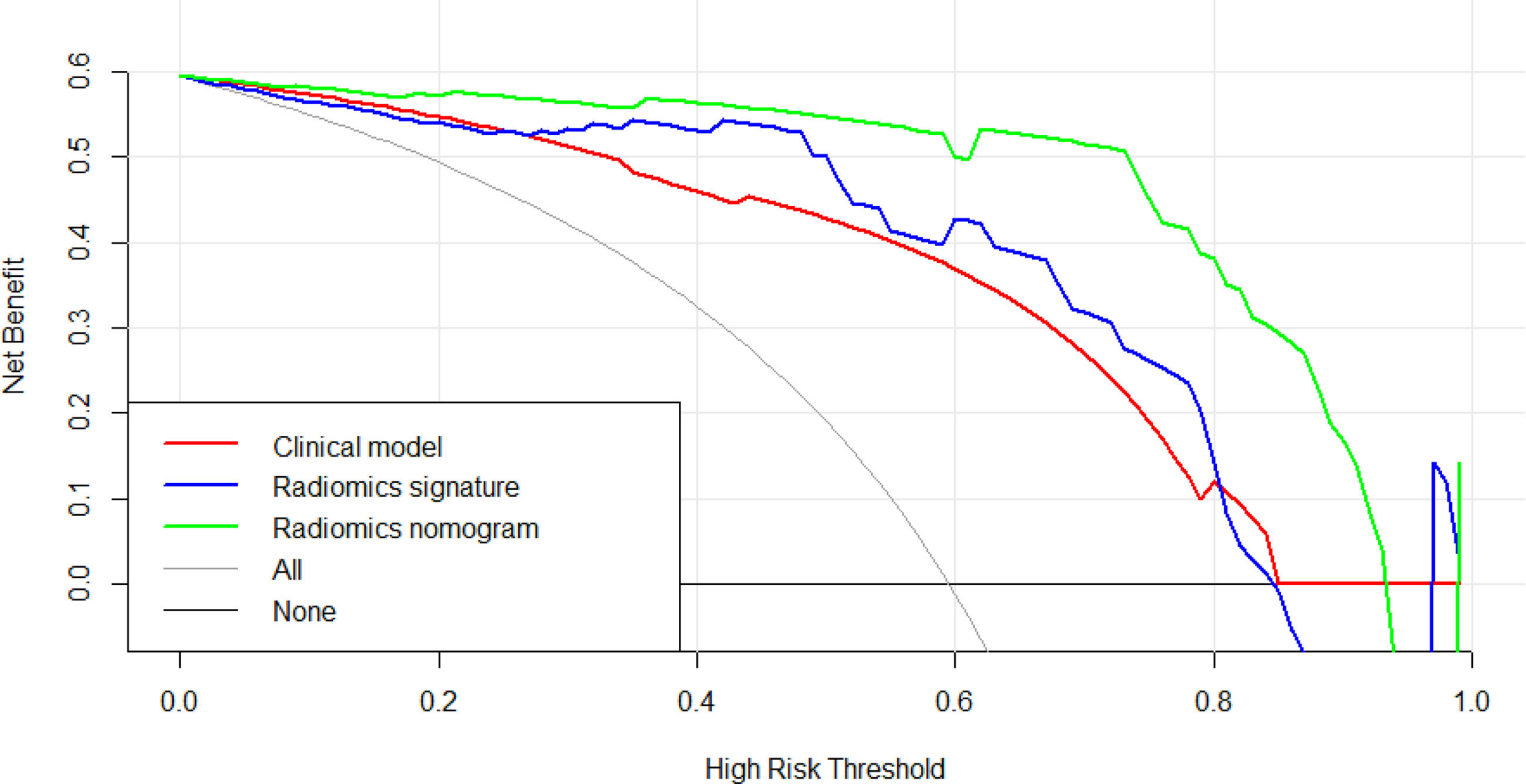
The decision curve analysis of CT findings model, radiomics model and radiomics nomogram in test cohort. The green line represents radiomics nomogram. The blue line represents radiomics model. The red line represents CT findings model. The grey line represents the assumption that all patients had pancreatic ductal adenocarcinoma (PDAC) (All). The black line represents the assumption that all patients had focal autoimmune pancreatitis (fAIP) (none). Threshold probabilities in clinical decisions take arbitrary values and patients will benefit more from the radiomics nomogram than either the treat-no-patient or treat-all-patient options.

The **Figure 9** presents the DCA curves. We observed that the patients would benefit more from the radiomics nomograms than either the treat-no-patient schemes or the treat-all-patients regimens. Furthermore, the DCA curve showed that the radiomics nomogram had a higher net benefit than the curvilinear CT discovery model and the radiomics model in identifying patients with PDAC.

## 4 Discussion

In the present study, we developed and validated a diagnostic radiomics nomogram model combining subjective CT findings and radiomic features as a novel and effective complementary method for preoperative identification of fAIP and PDAC. The calibration curve, ROC curve and decision curve were used to verify the discriminating efficacy of our model. All evaluation metrics show that the nomogram model outperforms the single model in distinguishing fAIP and PDAC, and the nomogram model enables model visualization. The nomogram model has potential as a decision tool for the need for surgical resection.

Some previous studies (21) found that some imaging features were more correlated with fAIP in contrast-enhanced CT than in PDAC. These included capsule-like rim with low attenuation (5, 22–26), without atrophic changes in uninvolved pancreatic tissue (23, 27), without MPD dilatation upstream (> 5 mm) (28), and our study showed similar results. Furthermore, we also found that the biliary wall thickening is helpful in differentiating the two diseases. This may be due to the fact that AIP is a systemic fibro-inflammatory disease, most commonly involving the bile ducts, resulting in sclerosing cholangitis (SC), biliary wall thickening and bile duct stricture (29); In PDAC however, there is only external compression, with rare cases of bile wall thickening. However, the diagnostic accuracy of imaging studies depends on the presence or absence of characteristic symptoms and the overall experience of the radiologist.

In recent years, radiomics techniques have rapidly developed, with the radiomics analysis aiming to provide a quantitative measure of intralesional heterogeneity. This is helpful in assessing tumor aggressiveness, treatment response and prognosis, and distinguishing benign from malignant lesions (29). The radiomics value in distinguishing between AIP and PDAC has been previously reported (21, 30–34). By extracting the radiomics features of the venous phase, Park et al (31) could distinguish AIP from PDAC with 89.7% sensitivity, 100% specificity, and 95.2% overall accuracy. The classification effect is better than that of the arterial phase, so in our study, the imaging data of the venous phase was used for the diagnosis of the two diseases. However, the previous study did not focus on fAIP patients, but included both diffuse AIP and fAIP patients. Furthermore, Zhang Y et al (30) and Liu Z et al (33) noninvasively classified PDAC and AIP lesions using PET/CT images using a radiomics-based predictive model. (Mean AUC: 0.9668, Accuracy: 89.91%, Sensitivity: 85.31%, Specificity: 96.04%). The above results show that establishing a radiomics signature model significantly improves the diagnostic efficiency.

To obtain an appropriate model able to distinguish between fAIP and PDAC, we developed and validated three models, and found that the combined nomogram performed better than the radiomics model and the CT findings model (training cohort AUC were 0.87, 0.73 and 0.67, and the test cohort AUCs were 0.83, 0.76, and 0.66). The calibration curves showed good agreement between the predicted values and the actual results. The decision curves showed that the radiomics nomogram model had a higher net benefit than the individual CT findings model and radiomics model respectively. By acquiring high-throughput quantitative features from CT images, radiomics signatures allow the assessment of tumor heterogeneity and the spatial distribution of biologically relevant voxels (9).

In our study, a two-step feature selection process screened 7 best features from 158 radiomic features, suggesting that these 7 features play a relatively important role in identifying fAIP and PDAC. For example, “LoG” (Laplace Gaussian) and “GLCM” (Gray Level Co-occurrence Matrix) are features that have proven useful in predicting the pathological features of certain tumor types (12, 35–37). We classified the filtered features using a variety of machine learning-based classifiers, and we chose these methods mainly because they were popular and performed well in previous studies (38). The performance of MLR classification is not the best on the training cohort, but it performed best on the test cohort. The performance of some classifiers in the training cohort and the test cohort is quite different. The preliminary judgment is that due to the problem of data volume and classifiers, RF and SVM were seriously overfitted. The radiomics features composed of the above 7 selected features are then represented by Rad-Score. When a patient has a high Rad-Score through CT image-based radiomic analysis, PDAC can be initially determined after comprehensive consideration. In addition, serum markers such as CA19-9 or IgG4 levels can be further detected, thereby establishing a personalized and convenient diagnostic system.

Histopathology obtained by endoscopic ultrasonography (EUS) guided fine-needle aspiration biopsy (EUS-FNA/B) is the gold standard for the AIP diagnosis. However, a recent multicenter study reported that the diagnosis rate for type I AIP using EUS-FNA/B was only 58.2% (39). EUS-FNB/B may not achieve definitive diagnosis even in the presence of large tissue volumes (40). The nomogram established in our study, combined with CT findings and radiomics features, is a non-invasive predictive tool that can analyze the overall characteristics of the lesion regardless of the location and size of the lesion. This may improve the accuracy of diagnosis, and reduce patient trauma with optimal compliance at the same time.

However, our study still has some limitations. First, CT images of fAIP patients were acquired over 11 years (2011 to 2021), whereas CT images of PDAC patients were acquired in the last 6 years (2017 to 2022). This may affect CT findings and features extracted. Second, due to the low incidence of fAIP, cases over nearly a decade have been included in our study, but there are still not enough cases to validate the proposed radiomics model, and selection bias is inevitable due to matched sampling. In order to verify the performance of our study on multi-center data, we initially selected CT image data of two patients from other hospital, and used the nomogram model to make predictions. The prediction results have been added to **Figures 7C, D**. It can be preliminarily seen from the prediction results that the nomogram model has good generalization ability and can be applied to new patient and multi-center data. However, the above-mentioned external verification data is seriously insufficient and has certain contingency. In follow-up study, we will continue to collect data and add more external validation data to enrich our study. And to overcome small and unbalanced sample size problems, the method maybe the future directions of our study which was used by Stefano Barone et al (41). Third, the contours of VOIs of pancreatic lesions may have some influence on the performance of our prediction model. In the current study, two radiologists manually delineated the contours of the lesions, and it is a time-consuming process. Therefore, methods requiring less manual intervention should be considered, and the establishment of automated pancreas segmentation software may help improve this situation. Automated segmentation is the development trend of lesion segmentation in radiomics (18, 42). But automated segmentation also has its disadvantages. Automated segmentation often requires a large amount of data for training, usually the methods used are based on deep learning, and in the current research status, automated segmentation can only identify disease with evident lesion areas. But for the identification of two types of diseases with complex lesion areas, the results of automated segmentation are often not appropriate. In our study, fAIP is a relatively rare disease, the amount of data is not enough to support the training of automated segmentation software, and the similarity between fAIP and PDAC is high, so some key features may be lost using automated segmentation. Therefore, we chose to use manual segmentation as the method of lesion segmentation. At the same time, to ensure the reproducibility of VOI, we selected two experienced abdominal radiologists to jointly segment VOI. But automated segmentation is still an important direction for our future studies, and we are continuing to collect relevant data to prepare for the construction of automated segmentation software. In theory, the only texture features that resulted to be reliable (ICC>0.75), could lead to the elimination of fundamental features for building the predictive model. This is indeed a limitation of our study, but it is already one of the best methods, and in our actual study, the reproducibility and quality of feature extraction are guaranteed due to the extensive experience of the physicians responsible for VOI segmentation.

In summary, we have developed a preoperative CT imaging-based radiomics nomogram for distinguishing between fAIP and PDAC with high accuracy and clear diagnostic value. Quantitative and noninvasive radiomics analysis may be a useful application to help clinicians develop personalized treatment plans.

## Data availability statement

The raw data supporting the conclusions of this article will be made available by the authors, without undue reservation.

## Ethics statement

The studies involving human participants were reviewed and approved by the ethics committee of the People’s Hospital of China Medical University. Written informed consent for participation was not required for this study in accordance with the national legislation and the institutional requirements.

## Author contributions

DL takes responsibility for the integrity of the work as a whole, from inception to published article, JL performed the research, collected and analyzed the data, completed the manuscript. NJ and YZ provided the technology support. All authors contributed to the article and approved the submitted version.
