# Supplementary material for: A CT based radiomics nomogram for differentiation between focal-type autoimmune pancreatitis and pancreatic ductal adenocarcinoma
Source: Front Oncol. 2023 Mar 1;13:979437. doi: 10.3389/fonc.2023.979437 (PMC10014827; doi:10.3389/fonc.2023.979437)
Supplement: Supplementary file 1 [file DataSheet_1.docx]

The parameters of LASSO algorithm:

• eps: 0.001

• alphas: [-0.001,0.05,50]

• fit_intercept: True

• normalize: True

• precompute: False

• max_iter: 100000

• tol: 0.0001

• copy_X: True

• cv: 5

• verbose: False

• n_jobs: 1

• positive: False

• random_state: 0

• selection: ‘cyclic’

The parameters of MLR:

penalty='l2',

solver='liblinear',

C=0.5,

max_iter=1000

The parameters of RF:

n_estimators=10000,

random_state=0,

n_jobs=-1

The parameters of SVM:

C=1.0,

kernel="RBF",

degree=3,

gamma = 1/7,

probablity=True,

max_iter=1，

random_state=0

The parameters of DT:

criterion=’gini’,

splitter=’best’,

max_depth=5,

min_samples_split=2,

min_samples_leaf=1,

min_weight_fraction_leaf=0.0,

max_features=None,

random_state=0,

max_leaf_nodes=None,

min_impurity_decrease=0.0,

min_impurity_split=None,

class_weight=None,

presort=False

LIFEx：

Spatial Resampling:

Spacing X (mm):0.74

Spacing Y (mm):0.74

Spacing Z (mm):1.0

Intensity Discretization:

Nb of grep levels:400.0

Size of bins:10.0

Intensity Rescaling:

absolute(bounds:min< >max)

min bound: -1000.0

max bound: 3000.0
